# ABO Blood Group and Dementia Risk – A Scandinavian Record-Linkage Study

**DOI:** 10.1371/journal.pone.0129115

**Published:** 2015-06-04

**Authors:** Senthil K Vasan, Klaus Rostgaard, Henrik Ullum, Mads Melbye, Henrik Hjalgrim, Gustaf Edgren

**Affiliations:** 1 Department of Medical Epidemiology and Biostatistics, Karolinska Institutet, Stockholm, Sweden; 2 Department of Epidemiology Research, Statens Serum Institute, Copenhagen, Denmark; 3 Department of Clinical Immunology, The Blood Bank, Rigshospitalet, University Hospital of Copenhagen, Copenhagen, Denmark; 4 Hematology Centre, Karolinska University Hospital, Stockholm, Sweden; Ehime University Graduate School of Medicine, JAPAN

## Abstract

**Background:**

Dementia includes a group of neuro-degenerative disorders characterized by varying degrees of cognitive impairment. Recent data indicates that blood group AB is associated with impaired cognition in elderly patients. To date there are no large-scale studies that have examined the relationship between ABO blood group and dementia-related disorders in detail.

**Methods:**

We used data from the SCANDAT2 database that contains information on over 1.6 million blood donors from 1968 in Sweden and 1981 from Denmark. The database was linked with health outcomes data from nationwide patient and cause of death registers to investigate the relationship between blood groups and risk of different types of dementia. The incident rate ratios were estimated using log-linear Poisson regression models.

**Results:**

Among 1,598,294 donors followed over 24 million person-years of observation we ascertained 3,615 cases of Alzheimer’s disease, 1,842 cases of vascular dementia, and 9,091 cases of unspecified dementia. Overall, our study showed no association between ABO blood group and risk of Alzheimer’s disease, vascular dementia or unspecified dementia. This was also true when analyses were restricted to donors aged 70 years or older except for a slight, but significantly decreased risk of all dementia combined in subjects with blood group A (IRR, 0.93; 95% confidence interval [CI], 0.88-0.98), compared to those with blood group O.

**Conclusions:**

Our results provide no evidence that ABO blood group influences the risk of dementia.

## Introduction

Dementia includes a heterogeneous group of disorders characterized by impairment of two or more areas of cognition such as memory, judgment, abstract thinking and higher cortical function, however not all individuals with cognitive impairment necessarily progress to frank dementia.[1] Both cognitive impairment and dementia are inter-related as they share several putative risk factors in common, including age, smoking,[2] chronic alcohol abuse,[3] obesity,[4] hypertension,[5] dyslipidemia,[6] and diabetes.[7] Recent evidences have highlighted the significance of certain common pathological mechanisms between both disease states.[8] A potential link that is increasingly recognized is an underlying vascular compromise to different brain regions resulting either in vascular damage with accompanying inflammation or a hypoperfusive ischemic cerebral infarct. Therefore, alterations in vascular factors are independent risk factors for both dementia [Alzheimer’s dementia (AD) and vascular dementia (VD)] as well as in cognitive impairment. [9,10]

A temporal relation exists between vascular compromise and onset of cognitive impairment or dementia. This is due to alterations in certain hemostatic factors such as fibrinogen, prothrombin factor, D-dimer and factor VIII [11–13], thereby increasing the likelihood of thrombosis and subsequent infarcts. Incidentally, these alterations in hemostatic factors that are involved in the coagulation pathway have also been shown to be associated with ABO blood group,[14,15] providing clues to a possible vascular pathology that links certain blood groups with risk of both cognitive impairment and subsequent dementia development.[16]

At present, little is known about the relationship between ABO blood group and risk of dementia. However, recently Alexander *et al*., using a case-control study design, demonstrated that blood group AB is associated with an increased risk of cognitive impairment in elderly subjects, providing evidence to the role of ABO blood groups in the deterioration of cognition and perhaps memory.[17] The close relationship between cognitive impairment and dementia, obviously allows one to suggest that a similar risk may exist also between ABO-blood group and dementia-related disorders. To our knowledge there has been only one small case-control investigation reported so far which failed to demonstrate any association between ABO and Alzheimer’s disease.[18] There are no data that has examined the ABO association with the entire spectrum of dementia-related disorders at a population level. We report the first comprehensive estimate of ABO blood group effects on dementia-related disorders using the large, bi-national Scandinavian donations and transfusions (SCANDAT2) database.

## Methods

The recently updated SCANDAT2 (Scandinavian donations and transfusions-2) database is a computerized, combined donation and transfusion register from Sweden and Denmark that includes information on >1.6 million healthy blood donors.[19] A detailed description of the database has been published previously.[20] For the current study, we included individuals who had donated blood at some point since 1968 in Sweden and 1981 in Denmark and followed them until emigration, death or end of follow-up (31st of December 2012), whichever appeared first. Using unique personal national registration numbers, the SCANDAT database was linked with nationwide population, death and migration registers, thus ensuring complete and unbiased follow-up of all study participants. Data on incident diagnoses of dementia types (AD, VD and unspecified dementia) and related co-morbidity (diabetes, stroke, myocardial infarction) were extracted using International classification of diseases (ICD) 8, 9 and 10 codes from the respective country’s inpatient, outpatient and cause of death registers allowing longitudinal assessment and follow-up of health outcomes.[21,22] The sensitivity of these diseases ascertainment using ICD codes have been reported previously to be very high. [23] Information on blood groups was extracted from donation registers that included actual record of ABO groups from clinical antigen testing at the time of blood donation.

### Statistical analysis

The relative risk of dementia in individuals with blood group A, AB and B compared to individuals with blood group O, expressed as incidence rate ratios (IRR), were estimated using log-linear Poisson regression models. We chose Poisson models because of its robustness when incorporating time-dependent factors for a large number of subjects. The analysis included two models i) adjusted for sex, country (Sweden/Denmark), attained age and calendar period of observation (the latter both in 1-year intervals) and ii) additional adjustment for co-morbidity. Subjects with unknown blood group and individuals who were not born in Sweden or Denmark were excluded from the analyses. Attained age and calendar period were treated as time dependent factors, allowing individuals to move between categories over time, and were included in the models as restricted cubic splines with 7 knots. The models also included an interaction between the country and calendar period. To test if the association between blood group and risk of dementia was confounded by donation activity adjustments for number of blood donations were included in a time dependent manner, using restricted cubic splines. 95% confidence intervals (CI) were constructed using Wald tests. Analyses were performed both overall and restricted to the attained age group of 70 years or older as common etiologies for vascular and Alzheimer dementia advances with age and pathologies accumulate over time [24]. All statistical analyses were performed using SAS (Version 9.4, SAS Institute, Inc., Cary, North Carolina). P-values < 0.05 were considered statistically significant. The study was approved by the regional ethics committees in Stockholm, Sweden and the Danish Data Protection Agency in accordance with national legislations.

## Results

After exclusion of donors not born in Sweden or Denmark [N = 105,788 (6%)] and donors with unknown blood group [63, 441 (3.6%)], a total of 1,598,294 donors with a valid identity and who had performed at least one whole blood, plasma or platelet donation were included for analysis. Of these 780,132 (48.8%) were women. The median age at entry was 29.3 years (interquartile range [IQR], 22.5–40.0) and the median duration of follow-up was 15 years (IQR, 8.4–22.9). Other baseline demographics of the study participants are presented in Table 1.

**Table 1:** Baseline characteristics of the study population.

No. Subjects at study entry, N	1,598,294
Female, N (%)	780,132 (48.8)
Blood type, N (%)	
A	706,516 (44.2)
AB	81,313 (5.1)
B	175,462 (11.0)
O	635,003 (39.7)
Median (IQR) age at start of follow-up	29.3 (22.5–40.0)
Median (IQR) year of first donation	1997 (1989–2003)
Median (IQR) duration of follow-up	15.0 (8.4–22.9)
Attained Age ≥ 70 years, N (%)	175 112 (13.1)
Country, N (%)	
Sweden	1,067,655 (66.8)
Denmark	530,639 (33.2)

During 24 million person-years of follow-up, we observed 3,615 cases of AD, 1,842 cases of VD, and 9,091 cases of all dementia combined, including both specific and unspecific dementia cases. In the overall analyses, we observed no significant associations between ABO blood group and risk of AD, VD or all dementia combined for both models as described previously (Table 2). Addition of parameters for number of blood donations did not modify the risk estimates for ABO blood group (data not shown). When the analyses were restricted to donors age 70 or older, a similar pattern was observed, albeit with a statistically significantly decreased risk of all dementia combined in subjects with blood group A (IRR, 0.93; 95% confidence interval [CI], 0.88–0.98). Adjustment for comorbidity in this age group also did not alter the risk estimates. We observed no statistically significant interactions between ABO blood group and attained age, calendar period, or sex (data not shown).

**Table 2:** Incidence rate ratio of Alzheimer's disease, vascular dementia, all dementia combined by ABO blood groups stratified according to attained age.

	All age groups				≥70 years			
	Events	Person years	IRR (95%CI)*	IRR (95%CI)**	Events	Person years	IRR (95%CI)*	IRR (95%CI)**
Alzheimer’s disease								
A	1522	10,659,316	0.96 (0.89–1.03)	0.95 (0.88–1.03)	1030	359,899	0.94 (0.86–1.02)	0.94 (0.86–1.02)
AB	213	1,254,351	1.02 (0.85–1.22)	1.01 (0.87–1.18)	154	47,738	1.05 (0.86–1.27)	1.04 (0.88–1.24)
B	438	2,663,233	1.06 (0.95–1.20)	1.06 (0.94–1.20)	291	94,332	1.03 (0.89–1.19)	1.02 (0.90–1.17)
O	1442	9,502,670	1.00 (Ref)	1.00 (Ref)	1000	329,874	1.00 (Ref)	1.00 (Ref)
Vascular dementia								
A	821	10,662,516	1.06 (0.88–1.27)	1.05 (0.95–1.17)	630	361,991	1.04 (0.92–1.19)	1.03 (0.92–1.16)
AB	101	1,254,848	0.99 (0.72–1.36)	0.99 (0.79–1.25)	76	48,054	0.94 (0.70–1.28)	0.93 (0.73–1.18)
B	219	2,664,221	1.10 (0.92–1.31)	1.10 (0.94–1.29)	162	94,945	1.05 (0.85–1.29)	1.06 (0.89–1.26)
O	701	9,505,482	1.00 (Ref)	1.00 (Ref)	547	331,576	1.00 (Ref)	1.00 (Ref)
All dementia								
A	3819	10,651,326	0.95 (0.90–1.00)	0.95 (0.90–0.99)	2546	355,570	0.93 (0.88–0.99)	0.93 (0.88–0.98)
AB	502	1,253,347	0.95 (0.81–1.11)	0.95 (0.86–1.05)	350	47,141	0.95 (0.83–1.08)	0.94 (0.84–1.05)
B	1100	2,660,830	1.06 (0.98–1.14)	1.05 (0.99–1.13)	728	92,969	1.03 (0.94–1.13)	1.04 (0.95–1.12)
O	3670	9,494,580	1.00 (Ref)	1.00 (Ref)	2493	325,149	1.00 (Ref)	1.00 (Ref)

IRR* and IRR** denotes adjusted incidence rate ratio. IRR (95%CI) represents incident rate ratio and 95% confidence interval and were estimated using Poisson regression adjusted for country, sex, attained age, calendar period of observation and the interaction between country and calendar period of observation for adjusted model-1*. In adjusted model-2**, the model included additional adjustments for diabetes, stroke and myocardial infarction. Analyses were restricted to subjects with known blood group who were born in Sweden or Denmark. Blood group O is the reference category.

## Discussion

Using information on blood donors from SCANDAT2 database, we demonstrate that ABO blood group is not associated with the risk of dementia of any type. This was also true when analyses were restricted to donors aged 70 years or older. The strengths of our study include the large, population-based study-design with access to data on most important comorbidities, use of nationwide registers with virtually complete and unbiased follow-up as well as the availability of prospectively recorded measurements of blood groups prior to disease occurrence, the latter largely excluding the possibility of any misclassification of the primary exposure.

Our study provides solid evidence of absence of even a relatively weak association between ABO blood group with clinical dementia among a healthy donor population followed for up to 44 years and is consistent with a previously published case-control study.[18] Recently Alexander *et al*., using REGARDS cohort showed a statistically significant association between blood group AB and cognitive impairment.[17] However, they discuss that this relationship is possibly mediated through the effect of ABO blood group on cardiovascular risk factors particularly hemostatic factors that are involved in the coagulation pathway.

Cognitive impairment is a pre-symptomatic state of dementia and it is noteworthy to point that two-thirds of individuals who qualify for clinically definable cognitive impairment do not develop dementia.[1] This is possibly because certain secondary causes of cognitive impairment (e.g., depression, side-effects of drugs, thyroid disease, and vitamin B12/folate deficiency) when treated do not progress to dementia. The ABO association with cognitive impairment and absence of association with dementia can be explained by the fact that i) the risk conferred by blood type on cognitive impairment might operate through different mechanisms which do not actually relate to clinical dementia; or ii) The REGARDS cohort included individuals with risk factors for stroke mortality who already might have a cumulative burden of cerebrovascular lesions that explain cognitive decline. In such situations it is possible, that multiple risk factors for stroke may interact synergistically, or be linked to the ABO blood groups and hence impair cognition. In line with the argument, it is intriguing to note that in the REGARDS cohort, the association with cognitive impairment was observed only with AB blood group, but not with A or B groups, which have been shown previously to relate to stroke.[25] In our study, the younger mean age of our cohort minimizes the possible effects of other comorbidities. Although we did not have access to detailed data on other risk factors such as smoking, alcohol consumption, hypertension or dyslipidemia it is likely that accounting for other confounding factors would have only a limited effect on our results given that these are not associated with ABO blood groups. This is also evident by our unchanged risk estimates when adjusted for underlying comorbidity such as diabetes, stroke and myocardial infarction, which indirectly account for the conventional risk factors as mentioned above.

The association between cognitive impairment and non-O blood group is possibly related to the increased levels of FVIII/vWF and other hemostatic factors. [26,27] Sustained elevated levels of such pro-thrombotic markers increase the risk for subcortical or lacunar infarcts leading to cognitive impairment among individuals with non-O blood group. Although, this pro-thrombotic mechanism may also partially explain the vascular pathology underlying VD and AD,[28] we were unable to demonstrate any significant risk of dementia with non-O blood group. Also, we are unable to explain the reason for significantly decreased risk of all dementia combined in subjects with blood group A in individuals aged ≥ 70 years; however we speculate that it may be a chance finding.

Some limitations need to be acknowledged. Dementia diagnosis was based only on register based outcomes data which includes diagnosis provided by a clinician and hence some degree of under-reporting or even outcome misclassification can be expected. However, dementia diagnosis in the Swedish patient register has previously been shown to have a reasonably high specificity and moderately high sensitivity.[29] It is unlikely that the any degree of misclassification would differ between blood groups and thereby affect the relative risk estimates. We additionally caution that the blood donors may not be fully representative of the general population with regards to prevalence of early signs of dementia as well as of certain risk factors for dementia as they are apparently healthy at the time of blood donation. As our study was aimed to investigating the effect of a genetic risk factor on the long-term occurrence of a prospectively ascertained outcome, we believe that this does not lead to selection bias. Also assuming that the ABO blood group may interact with some other risk factors, it is possible that the general healthiness of the donor population may affect the external validity / generalizability of the investigation, but not the internal validity.

## Conclusions

We conclude that, although progressive worsening of cognitive function is a clinical hallmark of dementia development, the association between ABO blood group and cognitive impairment does not necessarily relate also to dementia. We speculate that the ABO-blood group related pathological mechanisms that operate during early cognitive impairment might have a much lower impact compared to other conventional risk factors that predominate during later stages frank dementia.
